# A Label-Free CRISPR/Cas12a-G4 Biosensor Integrated with FTA Card for Detection of Foodborne Pathogens

**DOI:** 10.3390/bios15040230

**Published:** 2025-04-05

**Authors:** Anqi Chao, Qinqin Hu, Kun Yin

**Affiliations:** School of Global Health, Chinese Center for Tropical Diseases Research, Shanghai Jiao Tong University School of Medicine, Shanghai 200025, China; angelchao@sjtu.edu.cn

**Keywords:** CRISPR/Cas, G-quadruplex, FTA card, nucleic acid extraction, foodborne pathogen

## Abstract

CRISPR/Cas-based diagnostics offer unparalleled specificity, but their reliance on fluorescently labeled probes and complex nucleic acid extraction limits field applicability. To tackle this problem, we have developed a label-free, equipment-free platform integrating FTA card-based extraction, CRISPR/Cas12a, and pre-folded G-quadruplex (G4)–Thioflavin T (ThT) signal reporter. This system eliminates costly fluorescent labeling by leveraging G4-ThT structural binding for visible fluorescence output, while FTA cards streamline nucleic acid isolation without centrifugation. Achieving a limit of detection (LOD) to 10^1^ CFU/mL for *Escherichia coli* O157:H7 in spiked food samples, the platform demonstrated 100% concordance with qPCR and standard fluorescent probe-based CRISPR/Cas12a system. Its simplicity, minimal equipment (portable heating/imaging), and cost-effectiveness make it a revolutionary tool for detecting foodborne pathogens in resource-limited environments.

## 1. Introduction

Foodborne diseases caused by pathogens pose a significant public health challenge, imposing substantial healthcare and economic burdens [1]. According to the statistics, nearly 600 million people—almost one in ten—suffer from foodborne illnesses annually, resulting in an estimated 420,000 deaths [2]. Food safety risks can emerge at any point along the food supply chain from “farm to fork” [3], underscoring the critical need for rapid and accurate pathogen detection to enhance food safety and minimize associated risks.

Common foodborne pathogen detection techniques include plate cultivation [4,5], omics-based methods [6], immunological assays [7,8], and molecular diagnostics like quantitative polymerase chain reaction (qPCR) [9,10]. Although these techniques offer high sensitivity and reliability, their widespread application is hindered by limitations including lengthy processing times, high costs, and dependence on laboratory equipment and skilled personnel, making them less feasible for use in resource-limited environments [11].

Clustered regularly interspaced short palindromic repeats (CRISPR) and CRISPR-associated nucleases (Cas proteins)-based nucleic acid diagnostic has developed as a powerful molecular diagnostic tool due to its programmable nucleic acid recognition and *trans*-cleavage activity [7,12]. Upon recognition of a complementary target sequence by CRISPR RNA (crRNA), the associated Cas effector proteins become activated, enabling precise and efficient nucleic acid cleavage. These proteins cleave their specific target sequences through *cis*-cleavage while simultaneously exhibiting collateral *trans*-cleavage activity, indiscriminately cutting single-stranded DNA (ssDNA) reporters to generate visible signals [13,14,15]. By integrating with various signal amplification techniques and signal output modes, CRISPR/Cas technology has achieved significant breakthroughs in the field of diagnostics [16]. However, conventional CRISPR/Cas detection relies on labeled quenched-fluorescent ssDNA reporters, which require complex labeling and are expensive [17,18]. Additionally, current CRISPR/Cas-based foodborne pathogen detection requires labor-intensive sample pretreatment and nucleic acid extraction, often requiring commercial kits and specialized equipment [19]. Therefore, there is a growing demand for a CRISPR/Cas detection system with a label-free signal reporter and simplified sample processing.

Guanine quadruplexes (G4s) are non-canonical DNA secondary structures that arise from guanine-rich sequences through Hoogsteen hydrogen bonding in the presence of monovalent cations (such as K^+^ or Na^+^) [20,21]. Due to their ease of synthesis, exceptional thermodynamic stability, and selective ligand-binding capabilities, G4s have gained attention in biosensor development [22,23,24,25]. They can specifically bind to ligands like Thioflavin T (ThT), producing strong fluorescence signals without complex reporter modification [26,27]. Their structural stability, synthetic accessibility, and ligand-binding specificity make them ideal for integration into CRISPR-based biosensors, enabling cost-effective, equipment-free pathogen detection.

In this article, we established a label-free fluorescent CRISPR/Cas12a detection system integrated with G4 signal reporters and the Flinders Technology Associates (FTA) card-based nucleic acid extraction for rapid, sensitive identification of foodborne pathogens. By leveraging pre-folded G4 structures that bind ligands (e.g., Thioflavin T), we eliminated the need for complex fluorescent labeling, establishing a cost-effective and equipment-free detection strategy. To streamline field applicability, we introduced a simplified, equipment-free sample preparation method using FTA cards, allowing rapid and efficient pathogen detection. Our approach demonstrated exceptional sensitivity and accuracy in food samples, underscoring its potential for reliable, rapid, and cost-effective pathogen detection in complex food sample matrices.

## 2. Materials and Methods

### 2.1. Reagents and Materials

Target foodborne pathogen strain *E. coli* O157:H7 (CICC 10907) and other nontarget strains, including *S. typhimurium* (CICC 21483), *L. monocytogenes* (CICC 21635), *S. aureus* (CICC 10201), and *V. parahaemolyticus* (CICC 21617), were sourced from the China Center of Industrial Culture Collection. All oligonucleotide sequences (Table S1), comprising plasmid templates, primers, G4 sequences, and crRNA sequences were supplied by Tsingke (Beijing, China) or GenScript Biotech (Piscataway, NJ, USA). EnGen Lba Cas12a (Cpf1), and its corresponding reaction buffers were purchased from NEB (Ipswich, MA, USA). The TwistAmp Basic Kit, used for recombinase polymerase amplification (RPA) reaction, was obtained from TwistDx (Cambridge, UK). FTA cards (WB120204), along with FTA purification reagent, and TE buffer were procured from Whatman (Little Chalfont, Buckinghamshire, UK). Thioflavin T (ThT) was supplied by Sigma-Aldrich (St. Louis, MO, USA). Fast SYBR™ Green Master Mix was acquired from Applied Biosciences (Foster City, CA, USA). Phosphate-buffered saline (PBS) and other common chemicals were obtained from Sinopharm (Shanghai, China). Milk for spiked sample detection was purchased from the ALDI supermarket in Shanghai. Fluorescence signal detection was conducted using a QuantStudio™ 7 Flex Real-Time PCR System (Applied Biosystems™, Thermo Fisher Scientific, Waltham, MA, USA). The endpoint detection assay was carried out in a portable heating block provided by Coyote Bioscienc (Beijing, China), and the endpoint fluorescent images were recorded using Tanon MINI Space 2000 (Shanghai, China).

### 2.2. Bacteria Pretreatment and DNA Extraction

Bacterial cultures were grown in Luria–Bertani (LB) broth medium for 12 h (37 °C, 160 rpm). The standard plate culture method was employed to quantify bacterial populations. The bacterial solutions were then serially diluted with sterile PBS (pH 7.4) to create bacterial samples with different final concentrations ranging from 10^0^ to 10^5^ CFU/mL. Then, the bacterial culture solution was pretreated following the guidelines outlined in the Whatman™ FTA™ Cards manual. Specifically, 100 µL of the target bacterial sample was applied onto the FTA card surface. A 2 mm diameter disc was then punched and transferred into a microcentrifuge tube. The disc underwent sequential washing twice, once each with FTA purification reagent and TE buffer. It was then dried at 56 °C for 10 min or dried at room temperature (25 °C) and prepared for further analysis.

### 2.3. Preparation of Pre-Folded G4s

The G4 sequences were diluted with Tris-HCl buffer (100 mM pH 7.4) to 20 μM. The mixture was then heated to 95 °C for 5 min, and subsequently cooled throughout 2 h to room temperature (25 °C) to ensure proper structure formation.

### 2.4. Pre-Amplification System Coupled Label-Free Fluorescent Detection System

The target DNA was pre-amplified by RPA using TwistAmp Basic Kit (Cambridge, UK). Specifically, a 50 μL of RPA reaction mixture was prepared by combining the reaction pellet, rehydration buffer (29.5 µL), forward and reverse primers (2 µL, 240 nM), magnesium acetate (2.5 µL), and nuclease-free water. Either a small FTA disc containing extracted DNA or 4 μL of DNA plasmid template was introduced into the RPA mixture, which was subsequently reacted at 37 °C for 20 min.

The label-free fluorescent CRISPR/Cas12a detection system consisted of 100 nM LbCas12a, 100 nM crRNA, 1× NEB buffer, 1 μM pre-folded G4 structures, and 10 μM ThT. After amplification, 1.5 μL of RPA products were introduced to the CRISPR detection system and reacted at 37 °C in a real-time PCR system, with fluorescence signals recorded every minute. Endpoint fluorescent images were recorded under LED blue light.

### 2.5. Detection Performance in Spiked Food Samples

Pasteurized milk was purchased from ALDI supermarket. Pure culture of bacteria was introduced into milk samples to simulate contamination with final concentrations between 10^0^ and 10^5^ CFU/mL. The label-free fluorescent detection system described above was used for detection and analysis. Fluorescence signals were collected using a real-time PCR system and the endpoint fluorescent images were captured under LED light.

### 2.6. Detection in Quantitative PCR (qPCR) Method

Each qPCR reaction was prepared in a total volume of 20 μL, containing 2 μL of DNA template extracted from Trelief^®^ Bacteria Genomic DNA Kit (Beijing, China), 10 μL of Fast SYBR™ Green Master Mix (Foster City, CA, USA), and 0.5 μM forward and reverse primers. The thermal cycling program began with initial heating at 95 °C for 2 min, followed by 40 cycles comprising 95 °C for 15 s and 60 °C for 1 min.

### 2.7. Electrophoresis Analysis

The 20% native polyacrylamide gel (PAGE) was run in 1X TAE buffer at 200 V for 5 h. The gel results were recorded by Amersham™ ImageQuant™ 800 (Cytiva, Marlborough, MA, USA).

### 2.8. Statistical Analysis

GraphPad Prism 9.0 was utilized for data analysis, statistical significance, and visualization. Experimental results were reported with error bars as the mean ± standard deviation (SD), derived from 3 independent replicates. Statistical significance between groups was calculated using a two-tailed Student’s *t*-test with a *p*-value less than 0.05 considered statistically significant.

## 3. Results and Discussion

### 3.1. Overview Design of Label-Free Fluorescent CRISPR/Cas12a Detection System

To enhance detection sensitivity, nucleic acid isothermal amplification techniques such as RPA are often combined with CRISPR/Cas-based fluorescent reactions. This approach demonstrates significant potential for the rapid and accurate detection of foodborne pathogens. However, its application is typically limited by the complexity and high cost associated with double-labeled quenched-fluorescent reporters, as well as labor-intensive, time-consuming, and equipment-dependent sample pretreatment processes. Therefore, developing a label-free fluorescent RPA-CRISPR/Cas system integrated with a simplified pretreatment process is critical to advance its use for rapid and accurate detection of foodborne pathogens, particularly in resource-limited environments.

**Figure 1:**
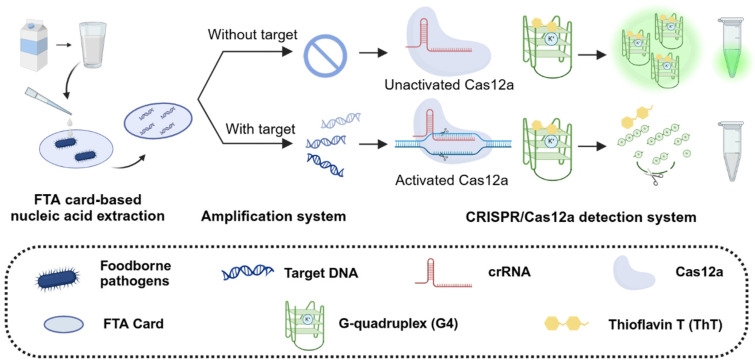
Schematic illustration of CRISPR/Cas12a-based label-free fluorescent detection system.

To tackle these challenges, we proposed a novel fluorescent detection system that integrates the CRISPR/Cas12a system with G4 signal reporter and FTA card-based nucleic acid extraction. This system represents a promising advance in foodborne pathogen detection methodologies. As shown in Figure 1, the detection process and principle of our system are as follows: pathogens in food samples are first processed using an FTA card membrane. This special filter paper is impregnated with special chemical denaturants and chelating agents, enabling bacterial lysis, protein denaturation, genomic DNA extraction, and DNA preservation against degradation [28]. This approach is simple to operate, enables rapid purification, and exhibits high nucleic acid extraction efficiency. Pathogens are first captured by the FTA card, and after a series of treatment steps—including bacterial lysis, impurity washing, and card drying—the FTA card serves as a template for subsequent RPA-CRISPR detection. This streamlined process significantly improves the efficiency of sample preparation while preserving the sensitivity and reliability of pathogen detection.

Due to its simplicity and high sensitivity, G4 (sequence shown in Table S1) was selected as a label-free reporter for the CRISPR/Cas12a system, according to the previous study [22]. This system leverages the interaction between the G4 sequence and Thioflavin T (ThT) to generate an initial intense fluorescence signal. In the presence of target foodborne pathogens, the target DNA undergoes pre-amplification in the RPA system, producing a large number of activators that bind to Cas12a and trigger the CRISPR system. Once activated, Cas12a exhibits collateral cleavage activity, rapidly cleaving the G4 sequence and disrupting its secondary structure, resulting in a significant decrease in fluorescence signals. Conversely, in the absence of target pathogens, no DNA amplification occurs, Cas12a remains inactive, and fluorescence intensity remains unchanged. These fluorescence signal changes can be captured in real-time PCR instruments or observed under LED blue light, providing a sensitive and visually accessible detection method.

### 3.2. Verification and Optimization of G4 Reporter

**Figure 2:**
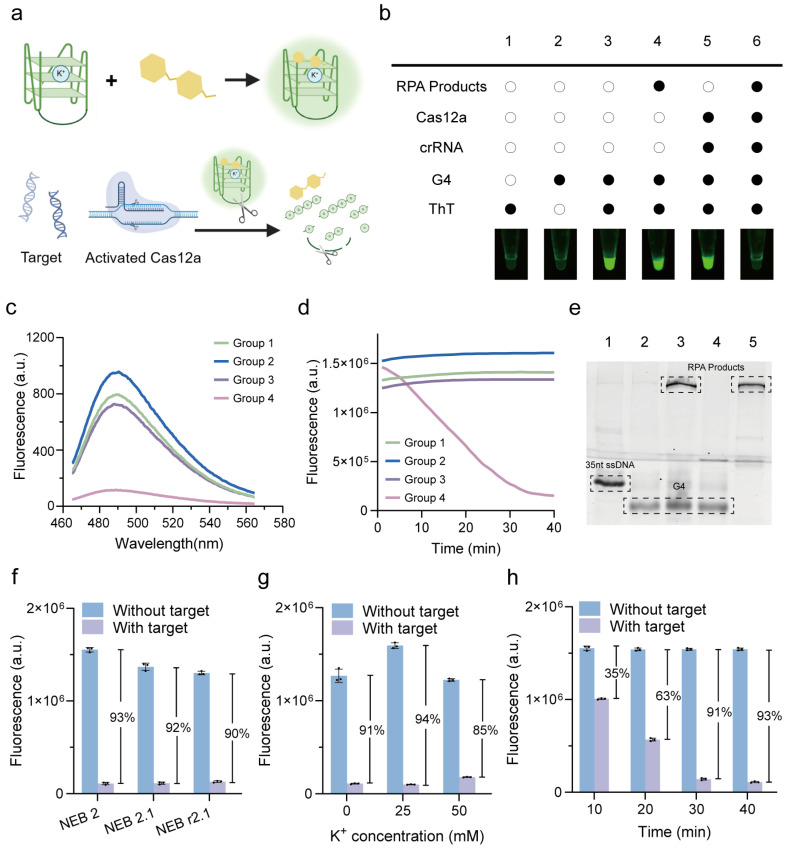
Verification and optimization of G4 reporter in label-free fluorescent CRISPR/Cas12a system. (**a**) Schematic representation of G4/ThT-CRISPR/Cas12a detection system. (**b**) Endpoint fluorescence images for analysis of G4/ThT and Cas12a activation. Fluorescence spectra analysis with excitation wavelength at 485 nm (**c**) and real-time fluorescence analysis (**d**) of G4/ThT complex. Group 1: G4-ThT. Group 2: G4-ThT-RPA. Group 3: G4-ThT-CRISPR. Group 4: G4-ThT-RPA-CRISPR. (**e**) PAGE assay of G4. Lane 1: 35nt ssDNA as a marker. Lane 2: G4. Lane 3: G4-RPA. Lane 4: G4-CRISPR. Lane 5: G4-RPA-CRISPR. Optimization of reaction buffer (**f**), concentrations of K^+^ (**g**), and reaction time (**h**) of CRISPR system, presented by decreased ratio of fluorescence between without target and with target. All error bars represent mean ± SD, where *n* = 3 replicates.

The G4-ThT complex generates a strong fluorescence signal, while activated Cas12a cleaves the G4 structure through collateral cleavage, leading to a significant decrease in fluorescent intensity (Figure 2a) [22]. To verify this principle, a series of reaction conditions were tested (Figure 2b). The combination of G4 and ThT alone (Group 3) produced a detectable fluorescence signal, confirming the intrinsic fluorescence response of the G4-ThT complex. Importantly, the addition of RPA products (Group 4) or CRISPR system components (Group 5), including Cas12a and its associated crRNA, did not interfere with the fluorescence of the G4-ThT complex, demonstrating the compatibility of the G4 reporter with the RPA or CRISPR system. However, in the presence of target DNA (Group 6), the G4-ThT fluorescence was significantly quenched, indicating that the CRISPR system was activated, cleaving the G4 sequence and disrupting its structure. This cleavage prevents the G4 sequence from binding to ThT, thereby suppressing fluorescence generation. Fluorescence and PAGE analysis further confirmed this principle. The presence of RPA products and CRISPR components exhibited significantly low fluorescence under 485 nm excitation, whereas other groups maintained high fluorescence levels (Figure 2c). The real-time fluorescence validates the stability of the G4-ThT complex over time and demonstrates the gradual decrease in fluorescence signal upon G4 cleavage (Figure 2d). The cleavage of G4 in the activated CRISPR/Cas12a system was further confirmed using PAGE assay (Figure 2e). Furthermore, a distinct signal difference was also observed between the RPA-negative control (no target in RPA) and the RPA-positive group (Figure S1). These findings validate the operational principle of the system, where the CRISPR/Cas12a-mediated cleavage of the G4 reporter serves as a sensitive and specific indicator of target DNA presence.

To optimize the CRISPR reaction, we investigated factors influencing G-quadruplex stability and fluorescence signal output. First, we evaluated the effect of the CRISPR reaction buffer (NEB 2, NEB 2.1, NEB r2.1, and their components can be seen in Table S2) and potassium ion (K^+^) concentration. As a result, the fluorescence signal decrease was most pronounced (97%) when using the NEB 2 buffer (Figure 2f), indicating its suitability for the reaction. As for the K^+^ concentration, the decreased ratio of fluorescence initially increased from 91% to 94% as the K^+^ concentration increased from 0 to 25 mM. However, a further increase in K^+^ concentration reduced the fluorescence quenching efficiency to 85%, indicating that 25 mM is the optimal K^+^ concentration for maintaining the G4 structure in the CRISPR reaction (Figure 2g). Additionally, we optimized the CRISPR reaction time before the signal readout. The fluorescence decrease ratio progressively increased from 35% to 91% as the reaction time was extended from 10 to 30 min. After 30 min, the signal reached a plateau, with no further significant changes observed (Figure 2h). These results indicate that a 30 min CRISPR reaction time is sufficient for complete cleavage of the G4 structure, maximizing fluorescence signal reduction and achieving optimal analytical performance.

### 3.3. Detection Performance of Label-Free Fluorescent CRISPR/Cas12a System with FTA Card-Based Pretreatment

Commercial nucleic acid extraction kits typically require large laboratory equipment such as high-speed centrifuges, limiting their applicability for rapid field testing in resource-limited environments [18]. In contrast, FTA card-based nucleic acid extraction offers several advantages such as simplicity, time efficiency, and reduced dependence on specialized personnel. In this study, the feasibility of integrating FTA card-based nucleic acid extraction into our label-free fluorescent CRISPR/Cas12a-G4 detection system was investigated.

To validate this integration, *Escherichia coli* O157:H7, a common Shiga toxin-producing *E. coli* (STEC) serotype, was selected as the model pathogen. The *rfbE* gene, specific to *E. coli* O157:H7, was used as the detection target. RPA primers and crRNA sequences were adapted from previous studies (Figure S2 and Table S1) [17].

**Figure 3:**
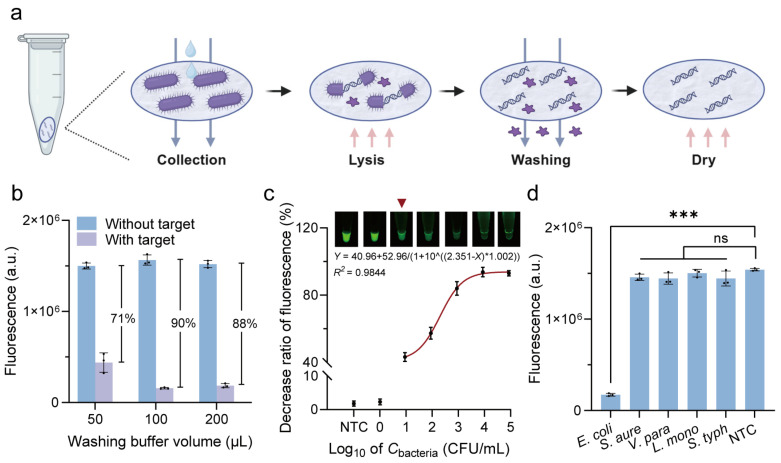
Detection performance of label-free fluorescent CRISPR/Cas12a system with FTA card-based pretreatment. (**a**) Procedures of FTA Card-based nucleic acid extraction. (**b**) Optimization of washing buffer volume of FTA purification reagent and TE buffer. (**c**) Calibration curve between decrease ratio of fluorescence and concentrations of *E. coli* O157:H7. Inset: Endpoint fluorescent images analysis. (**d**) Specificity analysis of label-free fluorescent detection system. *S. aure*, *Staphylococcus aureus*; *V. para*, *Vibrio parahaemolyticus*; *L. mono*, *Listeria monocytogenes*; *S. typh*, *Salmonella typhimurium*; NTC, no target control. All error bars represent mean ± SD from *n* = 3 replicates. Two-tailed *t*-test was used for significant comparison: *** *p* < 0.001; ns, not significant.

As shown in Figure 3a, bacterial cultures were pretreated according to the instructions for Whatman™ FTA™ Cards. After cell collection, lysis, washing, and drying steps, a 2 mm FTA card disc was punched and placed directly into the RPA system for amplification. The RPA products were subsequently added into the G4/ThT-CRISPR/Cas12a detection system. Results confirmed the successful integration of FTA-based extraction with our label-free fluorescent CRISPR/Cas12a detection system. The washing volumes of the FTA purification reagent and TE buffer were further optimized. As shown in Figure 3b, a washing buffer volume of 100 µL was optimal as it resulted in the maximal fluorescence signal decrease.

Under the optimized conditions, we assessed the detection performance of the label-free fluorescent CRISPR/Cas12a system with FTA card-based extraction method. To evaluate the sensitivity, *E. coli* O157:H7 strains with concentrations from 10^0^ to 10^5^ CFU/mL, as well as a no target control (NTC) containing only PBS, were applied to FTA cards for nucleic acid extraction. The fluorescence intensity progressively decreased with increasing concentrations of *E. coli* O157:H7 from 10^1^ to 10^5^ CFU/mL, and the corresponding regression equation between them was *Y* = 40.96 + 52.96/(1 + 10^((2.351 − *X*) * 1.002)) (*R*^2^ = 0.9844) (Figure 3c). A noticeable decrease in fluorescence was observed when the concentration of *E. coli* O157:H7 reached 10^1^ CFU/mL, indicating that the limit of detection (LOD) visible to the naked eye was as low as 10^1^ CFU/mL.

To assess the specificity, four common foodborne bacteria, specifically *S. aureus*, *V. parahaemolyticus*, *L. monocytogenes*, and *S. typhimurium*, were tested as non-target foodborne bacteria. Figure 3d showed a significant fluorescence decrease only for *E. coli*, whereas the signal remained relatively stable for the non-target foodborne bacteria, confirming high specificity. These findings demonstrate the potential of this system for the rapid and accurate detection of pathogens.

Furthermore, to verify the reliability of the amplification process and ensure that negative results arise from the absence or undetectable levels of the target rather than inadequate assay performance, we incorporated the *Neisseria gonorrhoeae porA* gene, a pathogen responsible for sexually transmitted infections, as the internal amplification control (IAC) into the G4/ThT-CRISPR/Cas12a system. Samples containing *E. coli* bacterial culture and/or *N. gonorrhoeae* DNA plasmid were applied to FTA card and subsequently used as templates for the RPA system, in which all reaction components were identical except for the primers. The RPA products were then introduced into the CRISPR detection system. The presence of the IAC enhances the reliability of the experimental results, as the presence of any target would result in a detectable change in the signal (Figure S3).

### 3.4. Application in Spiked Samples

**Figure 4:**
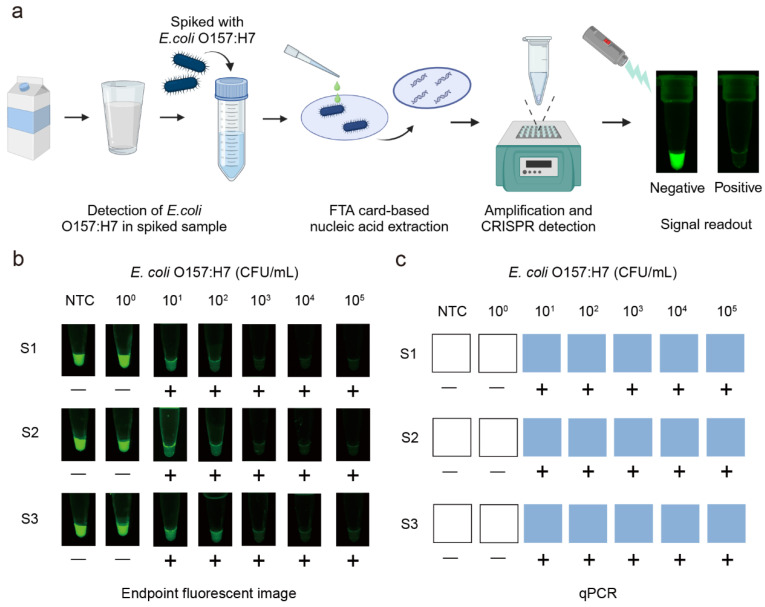
Detection of *E. coli* O157:H7 in spiked samples. (**a**) Workflow for detection of *E. coli* O157:H7 in milk by the label-free fluorescent detection system. (**b**) Endpoint fluorescence images under blue light. (**c**) Detection of *E. coli* O157:H7 in milk by qPCR. S1, S2, S3 represent replicates of samples. (−), negative; (+), positive; NTC, no target control.

To assess the performance of the label-free fluorescent CRISPR/Cas12a detection system in real food samples, we tested its ability to detect *E. coli* O157:H7 in pasteurized milk (Figure 4a). The pasteurized milk was inoculated with pure culture bacterial solution with varying concentrations between 10^0^ CFU/mL and 10^5^ CFU/mL. The spiked samples, as well as a no target control (NTC) containing only pasteurized milk, were processed using the integrated workflow, which included FTA card-based nucleic acid extraction, RPA reaction, and G4/ThT-CRISPR/Cas12a detection. To emphasize field applicability, we replaced laboratory equipment with a portable heating block for incubation and a blue LED transilluminator (λ = 485 nm) for fluorescence visualization. As shown in Figure 4b, endpoint analysis revealed a concentration-dependent fluorescence decrease, with visible signal quenching observed at bacterial concentrations as low as 10^1^ CFU/mL.

To evaluate the accuracy of the developed method, we compared the results with the gold-standard quantitative PCR (qPCR) method and standard fluorescent probe-based CRISPR/Cas12a system with FAM-BHQ labeled probes. The results obtained from the label-free fluorescent detection system (Figure 4b) were consistent with those from qPCR (Figure 4c) and the standard fluorescent probe-based CRISPR/Cas12a system (Figure S4), confirming the reliability and accuracy of our system for the foodborne pathogens detection in complex food matrices. Additionally, we compared the three methods in terms of sensitivity, detection time, and cost (Table S3). These findings highlight the potential of our system as a rapid, sensitive, and accurate diagnostic tool for the detection of foodborne pathogens in real food samples.

## 4. Conclusions

In this study, a cost-effective, label-free fluorescent detection system for foodborne pathogens was developed by integrating three key parts: (1) a pre-folded G4-ThT signal reporter to replace labeled probes, (2) FTA card-based nucleic acid extraction for equipment-free sample preparation, and (3) CRISPR/Cas12a-mediated target recognition for sequence-specific detection. The G4-ThT system enhances fluorescence signals through structural binding instead of covalent labeling, while FTA cards bypass centrifugation-dependent steps, enabling a fully field-deployable workflow. This system exhibits high sensitivity and specificity, achieving an LOD as low as 10^1^ CFU/mL in spiked milk matrices, with specificity confirmed against non-target pathogens. Validated against the gold-standard qPCR method and standard fluorescent probe-based CRISPR/Cas12a system, our system has a turnaround time of less than 1.5 h from sample to result using portable heating and imaging devices. By unifying sensitivity, simplicity, and cost-effectiveness, this approach addresses critical gaps in food safety monitoring, specifically in resource-limited environments where rapid on-site diagnostics are urgently required.

## Data Availability

The data are available upon reasonable request from the corresponding author.

## Supplementary Materials

The following supporting information can be downloaded at: https://www.mdpi.com/article/10.3390/bios15040230/s1, Figure S1: Comparison between RPA-negative (no target in RPA) and RPA-positive results. NTC, no target control. All error bars represent the mean ± SD from *n* = 3 replicates. Figure S2: The target sequence for the detection of *E. coli* O157:H7 using RPA primers and the crRNA sequence. The forward and reverse primers for the RPA reaction are shown in orange and green, respectively. The PAM sequence within the crRNA targeting *E. coli* O157:H7 (depicted in blue) is highlighted in red. Figure S3: IAC-incorporated detection. *E. coli*, *Escherichia coli*; *N. gono*, *N. gonorrhoeae*. All error bars represent the mean ± SD from *n* = 3 replicates. Figure S4: The detection performance of the standard fluorescent probe-based CRISPR/Cas12a system. NTC, no target control. All error bars represent the mean ± SD from *n* = 3 replicates. Two-tailed Student’s *t*-test was used for each two-group comparison: *** *p* < 0.001; **** *p* < 0.0001 ns, not significant. Table S1: Sequences of the nucleic acids used for detection in this study. Table S2: The components of CRISPR reaction buffer NEB 2, NEB 2.1 NEB r2.1. Table S3: The comparison of three detection methods.
