# Supplementary material for: A Label-Free CRISPR/Cas12a-G4 Biosensor Integrated with FTA Card for Detection of Foodborne Pathogens
Source: Biosensors (Basel). 2025 Apr 5;15(4):230. doi: 10.3390/bios15040230 (PMC12025128; doi:10.3390/bios15040230)
Supplement: Supplementary file 1 [file biosensors-15-00230-s001.zip › biosensors-3541574-supplementary.pdf]

Supporting Information

A label-free CRISPR/Cas12a-G4 Biosensor integrated with FTA Card for detection of Foodborne Pathogens

Anqi Chao [a], Qinqin Hu [a,*], Kun Yin [a,*]

[a] *School of Global Health, Chinese Center for Tropical Diseases Research, Shanghai Jiao Tong University School of Medicine, Shanghai 200025, China*

*Corresponding author

E-mail address: qinqinhu@sjtu.edu.cn (Q.H.); kunyin@sjtu.edu.cn (K.Y.)

Results and discussion

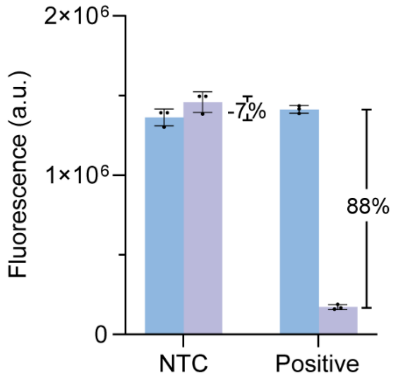

Figure S1. Comparison between RPA-negative (no target in RPA) and RPA-positive results. NTC, no target control. All error bars represent the mean ± SD from *n*=3 replicates.

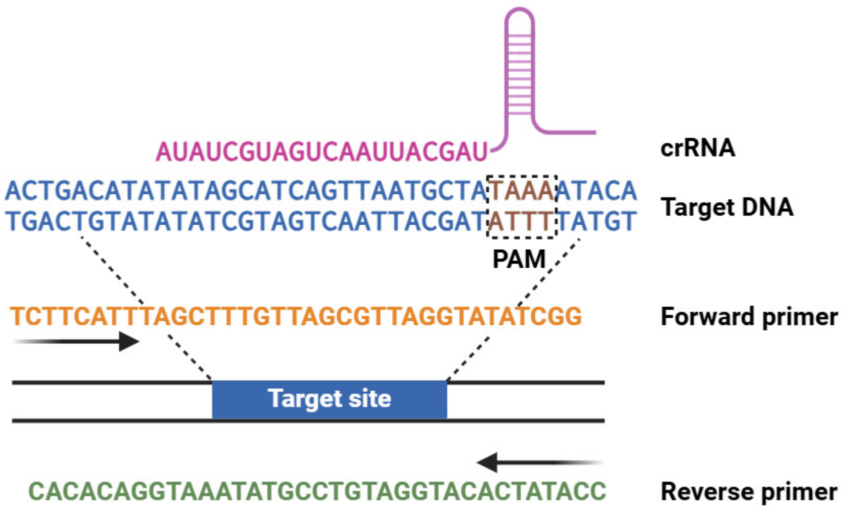

Figure S2. The target sequence for the detection of *E. coli* O157:H7 using RPA primers and the crRNA sequence. The forward and reverse primers for the RPA reaction are shown in orange and green, respectively. The PAM sequence within the crRNA targeting *E. coli* O157:H7 (depicted in blue) is highlighted in red.

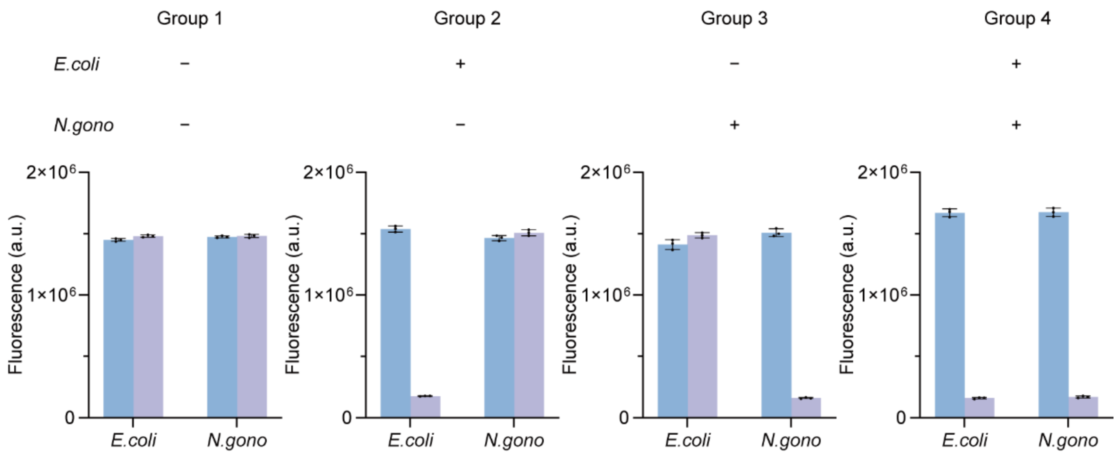

Figure S3. IAC-incorporated detection. *E. coli, Escherichia coli; N. gono, N. gonorrhoeae*. All error bars represent the mean ± SD from n=3 replicates.

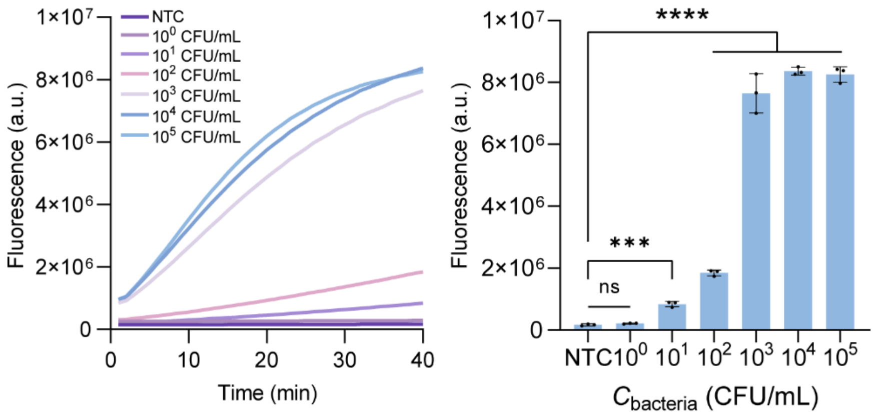

Figure S4. The detection performance of the standard fluorescent probe-based CRISPR/Cas12a system. NTC, no target control. All error bars represent the mean ± SD from n=3 replicates. Two-tailed Student's t-test was used for each two-group comparison: ***$P < 0.001$; ****$P < 0.0001$ ns, not significant.

Table S1. Sequences of the nucleic acids used for detection in this study.

Name	Sequence (5'-3')
rfbE_RPA_F	TCTTCATTTAGCTTTGTTAGCGTTAGGTATATCGG
rfbE_RPA_R	CCATATCACATGGATGTCCGTATAAATGGACACAC
rfbE_crRNA	UAAUUUCUACUAAGUGUAGAUUAGCAUUAACUGAUGCUAUA
G4	AGGGTTAGGGTTATTATTATTTAGGGTTAGGG
qPCR-F	CGCAGAGGAAAGAGAGGAATTA
qPCR-R	ACATTGGCATCGTGTGGA
porA_RPA_F	TATTTTCAAACGCCACGACGGTATGCCGGTTTC
porA_RPA_R	GAAAGTAGCAGGCGTATAGGCGGACTTGCTGTT
porA_crRNA	UAAUUUCUACUAAGUGUAGAUGACUCGGAACAAAUUGAAUGCUG
ssDNA reporter	FAM-TTATT-BHQ1

Table S2. The components of CRISPR reaction buffer NEB 2, NEB 2.1 NEB r2.1.

NEB 2	NEB 2.1	NEB r2.1
10 mM Tris-HCl	10 mM Tris-HCl	10 mM Tris-HCl
10 mM $MgCl_2$	10 mM $MgCl_2$	10 mM $MgCl_2$
50 mM NaCl	50 mM NaCl	50 mM NaCl
1 mM DTT	100 µg/ml BSA	100 µg/ml Recombinant Albumin
pH 7.9@25°C	pH 7.9@25°C	pH 7.9@25°C

Table S3. The comparison of three detection methods

	G4/ThT - CRISPR/Cas12a system	qPCR	standard fluorescent probe-based CRISPR/Cas12a system
Sensitivity	10^1 CFU/mL	10^1 CFU/mL	10^1 CFU/mL
Detection time	45 min	120 min	45 min
Cost	~$2.0	~$1.0	~$5.0
